# The Book World of Medicine and Science

**Published:** 1899-08-19

**Authors:** 


### The Book World of Medicine and Science.

The Pharmacopœia of Guy's Hospital. Compiled by a Committee of the Staff. (London: H. H. G. Grattan. 1899.)

To all who are or who have been connected with Guy's Hospital the appearance of a new edition of the Guy's Pharmacopœia is a matter of considerable interest, and in every part of the world we have no doubt that Guy's men will be found comparing the new edition with the old, and noting how the favourite formulæ are getting on, regretting, maybe, sometimes the changes which of necessity have had to be introduced into the mixtures to which they have been accustomed from the days of their youth. The present edition is an admirable work, combining, as it does, with the formulæ special to Guy's all that is practically useful in the British Pharmacopœia, so far, that is, as prescribing is concerned, and we have no doubt that it will be found useful as a guide to prescribing by many besides Guy's men. It has evidently been thoroughly overhauled, and take it altogether it really is, in its new dress, a very modern little book. As a means of bringing out the real beauties of asafetida we should think that the Mist. Asafetidæ Effervescens, touched up as it is with ammonia and valerian, and with its froth made durable by the addition of mucilage, must be most efficacious. We notice that the old soda and rhubarb powder is now sweetened with gluside. This is rather a wrinkle, as the sweetness of powdered saccharin hangs about the mouth for a considerable time—let us hope until the rhubarb is forgotten.

An Epitome of the History of Medicine. By Roswell Park, A.M., M.D. (Philadelphia: F. A. Davis Company. 1899. Large octavo, pp. 370.)

The history of medicine is one of the most fascinating of studies, not only to the physician but to the layman as well. We may profitably ruminate over the long struggle of the men of old in their search for a sound pathology; we can still join in the symposia at the Alexandrian Museum and hear Euclid make his celebrated remark about the royal road which Ptolemy had found to anatomy; we may sigh over the sad fate of Serveus, and may rejoice that it was left to an Englishman to make the greatest discovery of modern medicine. To Dr. Park of Buffalo we are indebted for once more presenting us with it all, and for presenting it in a most readable form. In his preface we find mentioned the various fountains at which he filled his pitcher; and it is rather curious to notice that he does not seem to have heard of Withington's fine Medical History, published in 1894, which covers much of the ground of this work, except that Withington stops at the eighteenth century, whereas Park brings his history down to the nineteenth, and not a few graceful remarks are made of our English physicians and surgeons of the present day. Renouard's arrangement of the subject is followed, and it has certainly the merit of fairly clear subdivisions. We have the Primitive Period, ending with the Fall of Troy; the Mystic Period, ending with the dispersion of the Pythagoreans; the Philosophic Period, covering the time between that and the year 320 b.c.; the Anatomic Period comes next, and ends about 200 a.d.; the Arabic Period; the Erudite Period of the fifteenth and sixteenth centuries; and finally the Reform or Present Period. All these are carefully worked out in the 350 well-printed pages of the book. Personally we should have liked to see greater prominence given to the Arab Period, and more especially to that wonderful part of the palmy days of the Western Caliphate. In Cordova alone there were seventeen universities and seventy public libraries. It is said that at least a million of volumes were destroyed after the defeat of the Arabs by Ferdinand of Castiles. Had we those volumes now, what might they not have taught us! Dr. Park again digs up for us the old fossil about the burning of the Alexandrian Library by the Caliph Omar in 640, but he wisely appends a footnote to the purport that this statement of the priest-physician Abulfaragins has been vigorously denied. When reading these pages we cannot help being struck by the fact that medicine has suffered immensely from the respect paid to blind authority, and in looking at those mistakes of the past we should try to avoid similar ones in the present. While it may be assumed that the few students among us will ever revert to the classic works on medical history, it is all too certain that the busy practitioner cannot spare time for such an undertaking, and to the large mass of readers we strongly recommend this book and that of Withington. From both of these an extensive knowledge may be obtained. Dr. Park's studies in ancient medicine have brought him in contact with the numerous modifications of the old sun worship, and in his last chapter, which he names Iatrotheurgic Symbolism, he gives us a short essay on the subject which, although it may not have much to do with the rest of the book, is of perennial interest to us all. We venture to suggest to Dr. Park that he should enlarge the essay and publish it separately.

The Commonwealth of the Body. By A. Hawkins-Ambler, F.R.C.S. (London: The Scientific Press. 1899. Pp. 43. Price 1s. 6d.)

The substance of this little book has already appeared, we learn, as a series of articles in the *Leeds Mercury*. In its present form it is, Mr. Ambler tells us, intended as a reader for schools. But, pellucid as the style is, and simple the matters spoken of, it will, we trust, be read to advantage by children of a larger growth. Mr. Ambler does not profess in these pages to teach a science, but he has compressed within them a vast amount of simple information in a way that will be easily grasped by those who most lack it. Mr. Ambler has a felicitous knack of expression. But he makes no sacrifice of accuracy and never lapses into colloquialism. The sections on "Sight and Hearing" (by Mr. Russ Wood) are not less well done than others of the book. Works like these in the first place are of advantage to the body physical, but they are not without benefit to the body politic.

Golden Rules of Psychiatry. By Jas. Shaw, M.D. Golden Rules Series No. V. (Bristol: John Wright and Co. Price 1s.)

This little volume is written on somewhat different lines from the other volumes of the series. It is more systematic, a more complete review of the subject with which it deals, a less jerky series of disconnected aphorism. Whether it will any better than the others suit the taste of its readers we cannot tell, for we have not yet been able to guess even who are the readers of these tasty little volumes; but this we can say, that for its size it gives a curiously complete sketch of the subject on which it treats.

### NOTES ON BOOKS.

A little book has been published by Dotesio and Todd, of Lowestoft, on Lowestoft as a winter health resort, which gives us a short account of the attractions of this town and its neighbourhood. Special emphasis is laid on the winter characteristics of the place. "Just when most of the visitors have left Lowestoft to return to London and their suburban homes, to dull skies and muggy atmospheres, rain and chilly fogs, just then Lowestoft is lit up with sunshine and has autumn days as bracing as spring, sea colours such as it never has in summer, and dry air like a tonic." Which is no doubt true.

